# An alternative numerical approach for an improved ecological model of interconnected lakes with a fixed pollutant

**DOI:** 10.1007/s40314-023-02191-3

**Published:** 2023-01-23

**Authors:** Nilay Yönet, Burcu Gürbüz, Aytül Gökçe

**Affiliations:** 1 Graduate School of Science and Engineering, Yıldız Technical University, 34220 İstanbul, Turkey; 2 Institute of Mathematics, Johannes Gutenberg-University Mainz, 55128 Mainz, Germany; 3 Department of Mathematics, Ordu University, 52200 Ordu, Turkey

**Keywords:** Lake contamination problem, Pollution model, Numerical approach, Taylor polynomials and series, Model simulation, 37N25, 37N30, 65L05, 65L70

## Abstract

There is currently an undeniable demand for solutions to environmental issues, especially water pollution. Water is essential for life and lakes constitute a big portion of water sources. In this study, we introduce a modified numerical approach to a dynamic ecological model focused on a lake pollution problem. The model includes three connected lakes with certain parameters and unknown functions such as pollution quantities and lake volumes. First, a preliminary mathematical analysis of the variables of each lake is presented taking into account the system components and parameters. Then, we present our numerical approach considering a series expansion to approximate the problem with the help of the truncated Taylor series. We describe a convergent technique, and finally, demonstrate the numerical simulations of the approach for the different unknowns with appropriate parameters. According to the results, the application of our alternative approach to the lake pollution problem is successful in terms of producing highly accurate information outputs about pollutant quantities a better approximation than the previously studied numerical approaches for the unknown functions of time. Furthermore, it is applicable to other similar ecological and environmental dynamic systems, and to related fields.

## Introduction

Pollution in water, air, and soil are major environmental issues nowadays. Combined together, they cause even bigger problems. In recent years, humanity has struggled and is still struggling against a deadly pandemic, beside other diseases, global warming, droughts, floods, etc. whose predominant reason is of human origin (Alimonti et al 2022). The lockdown decision of governments, as a consequence of COVID-19, caused a significant reduction in human activities. Hence, the impact of the COVID-19 lockdown on the environment proved that the problematic environmental situation can be reversed (Silva et al 2021; Yang et al 2021). Especially, the fact of decreased water pollution during the lockdown period around the world (Manoiu et al 2022) is giving hope for a cleaner aquatic environment.

Decreased industrial and transportation activities have improved water quality besides reducing the emission of air pollutants, and greenhouse gases, globally. Decreased deforestation and fires, and regenerated populations of endangered species have been observed (Silva et al 2021). Aside from environmental restoration, there is still a negative impact of COVID-19 on the environment, especially plastic waste pollution because of personal protective equipment and medical waste, and also increased use and disposal of disinfectants and medicinal chemicals (Silva et al 2021; Yang et al 2021). The current level of plastic pollution negates the environmental benefits of the lockdown. Greenhouse gases released during the production of plastic materials, and the accumulation of those plastics in aquatic and terrestrial environments contribute to climate change and disruption of ecosystems (Silva et al 2021; Ford et al 2022; Thushari and Senevirathna 2020).

**Fig. 1:**
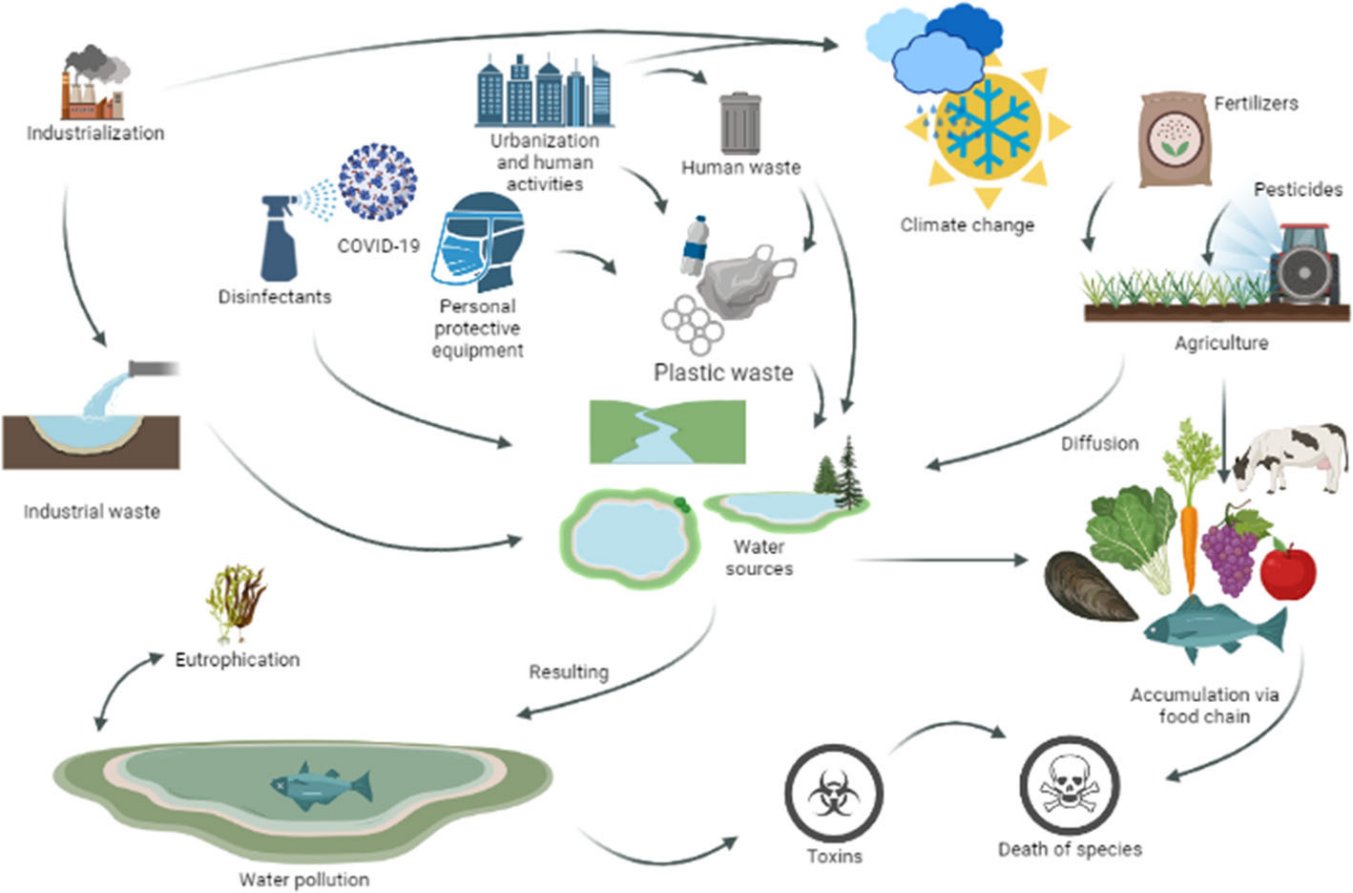
A simplified diagram illustrating parameters related to water pollution and how it affects life

Industrialisation (Nasrollahi et al 2020), urbanisation (Armeanu et al 2021), pollution (Persico and Johnson 2021), climate change(Ford et al 2022), global warming (Alimonti et al 2022), ecosystems (Bergstrom et al 2021), natural disasters (Fang et al 2018) including pandemics (Yang et al 2021), and physical (Eguiluz-Gracia et al 2020) and mental health (Marazziti et al 2021) are all related. In Fig. 1, different parameters affecting water pollution are shown with just basically directed interactions. Among many parameters, industrial waste introduces heavy metals, organic solvents, harmful chemicals, etc. Industrial waste and industrial activities also contribute to global warming and climate change, which are not the focus point of this study, through the hot wastewater, its vapor, and other greenhouse gases (Qadri and Bhat 2019). Along with industrialisation, urbanisation and human activities are other sources of water pollution (Chakraborti and Shimshack 2022). Domestic water usage returns human wastes along with chemical cleaning agents such as detergents (Qadri and Bhat 2019). In the past decades, plastic products as one of the most produced materials became essentials of our daily lives. Starting from their production, until the end of their lifetime, they form wastes (Geyer 2020; Daily 2021). With the pandemic, the usage of chemical cleaning agents and plastics, under the name of personal protective equipments, increased as well and added to the water pollution (Manoiu et al 2022).

Agricultural products are the products expected to be the purest. Fertilisers are the source of nitrogen and phosphorus which are necessary for the nutrition of plants. However, excessive fertiliser causes these nutrients to diffuse to the water sources through the soil. This situation does not look like a problem, but increased nutrient concentration forms a suitable environment for dominant algal blooms, sometimes resulting in eutrophication, and changing the balance of aquatic life. Pesticides for agricultural use, on the other hand, either cannot be metabolised by the plants or can diffuse to the water. So a wide range of toxins, joined by agriculture and marine products, accumulates in each step of the food pyramid and causes a wide range of diseases for living organisms (Fig. 1) (Qadri and Bhat 2019).

Water sources are especially important for all living organisms. Their pollution directly threatens all living beings, as mentioned earlier, and requires careful management (Qadri and Bhat 2019). Two important water sources are surface water, which is comprised of rain falling on the soil, and groundwater, which is formed due to the absorption of rain into the soil. Among these two water types, surface water sources such as rivers, lakes, and dams, have a high contribution to the domestic, agricultural, and industrial water demand (Hairom et al 2021; Sasakova et al 2018). As a member of surface water sources, lakes are water bodies surrounded by land. These water bodies do not have in and out-flows or they have very slow flows. Because of this stillness (nonflowing, lentic character), it is not easy to classify lakes and wide regions of rivers with very low flow rates. Due to the essentiality of water, lakes also became the center of life, civilisations, and economy throughout history (Dodds and Whiles 2020).

As surface water sources provide a big portion of the drinking and domestic usage water for our daily life, and lakes constitute a remarkable part of surface water. This is why we chose to model the pollution problem in lakes. In this work, we present an alternative numerical approach with the goal of contributing to studies on the environment, health, and economy in a roundabout way. The approach includes numerical techniques used to define problems for dynamical systems related to biological, chemical, physical, industrial, and economic fields, and focuses on the problem as a dynamical system comprised of three interconnected lakes with a source of contamination.

## Background and motivation

### Water pollution in terms of Lakes

Water sources are exceedingly sensitive to pollution (Hairom et al 2021; Sasakova et al 2018). Water quality has improved nearly all over the world during the COVID-19 lockdown period (Yang et al 2021). For instance, in shallow ground waters of South India, heavy metal and microbial concentrations decreased after the lockdown (Aravinthasamy et al 2021). River water quality has improved with the lockdown both in Turkey (Tokatlı and Varol 2021) and in China; in China, the water quality returned to its previous normal following the end of the lockdown (Liu et al 2022). Water quality in Vembanad Lake, India, is improved due to the decreased suspended particulate matter concentrations with the lockdown period (Yunus et al 2020).

As mentioned before, plastic pollution, medical waste, and, abuse of disinfectants, including hazardous chemicals, are consequences of COVID-19, and they threaten the cleanness of water sources and aquatic life (Silva et al 2021; Yang et al 2021). For instance, in Lake Tana, Ethiopia, pollution caused by personal protective equipments including surgical and reusable cloth masks and gloves threatens the lake ecosystem species with entanglements and ingestion of these fabrics. Additionally, the release of chemical agents and microplastics’ release from these personal protective equipments is another extent (Aragaw et al 2022). Even though COVID-19 increased it, plastic pollution was already a big issue as shown by studies monitoring, managing, and working on solving this pollution problem for surface water sources and aquatic and, coastal life (Ford et al 2022; Thushari and Senevirathna 2020). As reported in Driedger et al ’s review in 2015, plastics represent the biggest amount of pollution in the shorelines of Laurentian Great Lakes, North America, and plastic content of lake sediments is unknown. Paraná River in South America has a floodplain region containing thousands of lakes. The research (Blettler et al 2017) performed in 2017 has revealed the macro-, meso- and microplastics accumulation on the shorelines around one of the biggest of those lakes, Setúbal Lake, and has drawn a plastic pollution profile for all lakes in this region.

Similarly to the factors given above, the causes of lake pollution are commonly human activities. Industrial and sewage wastewater discharge possibly contains domestic waste, pathogens, heavy metals, and toxic pollutants. Fertilisers used in agriculture diffuse into the soil and then get transmitted to water. With the purpose of nutrient supply for agricultural plants, fertilisers are one of the most important sources of nitrogen- and phosphorus-based pollution in surface water resources including lakes (Hairom et al 2021; Sasakova et al 2018).

The nitrogen- and phosphorus-rich waste discharges create a nutrient-rich medium for photosynthetic organisms and induce the growth of the macroalgae population. This massive plant biomass prevents the transmission of sunlight through the depths of lakes, and adaptive photosynthetic species dominate the area. Also, some microbial species feeding on dominant species consume dissolved oxygen. Resulting hypoxia can reach lethal levels for other members of aquatic systems to sustain their lives. Low oxygen content, an increase in the microbial population, a decrease in biodiversity, and a reduction of light transmission in the lake convey eutrophication. Eutrophication-related microbial species, including harmful algal blooms, release toxic metabolites, for example, cyanotoxins. These toxins cause more pollution and can be metabolised by other members of the related ecosystem. Hence, the toxins can harm other organisms and participate in the food chain via bioaccumulation (Le Moal et al 2019). Another symptom of eutrophication is mucilage events. Mucilage is secreted by some eutrophication-related microorganisms, even though its production mechanism is not entirely known. Excessive mucilage aggregation forms a more suitable environment for microorganisms and contributes to eutrophication. An example of mucilage events is the one in the Marmara Sea in spring 2021, which resulted in massive deaths in marine life (Balkis-Ozdelice et al 2021).

Another human-sourced reason for surface water pollution is mining, which increases heavy metal concentrations in water (Hairom et al 2021) For instance, mercury used during gold mining accumulates as methylmercury and this accumulation is five to seven times greater in lentic water bodies, lakes, than rivers (Gerson et al 2020).

Pollution in water affects the health of human and aquatic ecosystems. The wealth of humans is also in danger because of water pollution’s negative influence on the economy. Economically, socially and politically pressured human populations despair even more, and environmental awareness expectation becomes impossible (Chakraborti and Shimshack 2022). Every little effort to improve the situation should be counted. Here, we aim to contribute to the research on pollution of water sources and environmental solutions. Furthermore, stagnant water bodies are more vulnerable to eutrophication and heavy metal accumulation. These facts raise the statement that lakes are more susceptible to pollution in various aspects than unstill water sources such as rivers. In this study, we chose to model the pollution of an interconnected three lakes dynamic system. In general, we believe that our study will aid the following endeavours about contamination of other water sources or other dynamic systems: similar environmental and ecological issues, and interdisciplinary studies including anthropic fields such as economy and health.

### Current solutions for water pollution in Lakes

Among all sources of the world, water is one of the worst preserved natural resources (Sasakova et al 2018). Water pollution, especially plastic pollution in lakes induced by COVID-19, is a crucial concern that needs to be addressed, and its awareness should be expanded, urgently (Aragaw et al 2022). Enhancement of water pollution management strategies would also help other environmental issues (Ford et al 2022).

The key measures that could be put in place are the identification of pollution sources, the reduction of pollutant emissions, and the promotion of recycling. Wastewater treatment is the primary solution strategy. However, there is still room for improvement. Wastewater treatment facilities should be supported and can be increased in number and areas. Treatment procedures could be specialised according to wastewater type or content. Additionally, there are sustainable, energy-efficient, thus economically feasible, environment-friendly, and still progressing bio-nanotechnology approaches (Yang et al 2021; Kakade et al 2021).

In the digitalised era, knowledge is the strength. Data collection and monitoring provide the basis for the understanding of water pollution. Furthermore, the correlations of climate change-related and anthropic parameters to water pollution and other parameters such as phosphorus, nitrogen, heavy metals’ concentrations, micro- and macroplastic content, temperature, pH changes, and the interrelationship with each other are not known entirely. Nonetheless, separately or collectively, many pieces of research are conducted all around the world. For example, concentrations of physical and chemical pollutants, specifically radioactive compounds and heavy metals, accumulated in marine sediments and could affect human health, have been measured (Caridi et al 2018). Monitoring these concentrations can constitute a data source to be used in different models, such as the fractional-order advection-dispersion-reaction (fADR) model. This model was used to study the dynamics of dissolved heavy metals and their transfer between the river and the riverbed (Puckett et al 2019). This type of model is significant for interpreting the physical and geochemical processes related to water pollutants. Another interesting example would be the research performed on the microplastic release of fishing nets (Montarsolo et al 2018). This research is also significant regarding microplastic absorption into the food chain and, by extension, its impact on human health. By contrast, microplastics are used consciously for pollutant adsorption in sewage treatment, and adsorption technology combined with microplastics is improved increasingly in other environmental treatment applications (Zhao et al 2022). Adsorbent materials with a recyclability property for the removal of organic pollutants also exist (Singh and Vaish 2019). Phosphorus is one of the most well-known contaminants that must be handled since it can enter water sources through residential wastes, soil due to agricultural fertilisers, and other channels. Phosphorus is a dangerous contaminant because it affects several factors, impacting aquatic life in diverse ways. A mathematical model (Tiwari et al 2021) has proved the efficiency of Phoslock, a phosphorus-locking technology, and is a promising method to explore the efficiency of different lake pollution treatment systems.

This wide range of examples reveals the need for collaboration in biochemistry, physics, mathematics, engineering, and more. However, the amount and variation of existing collective data on water pollution from these separate studies are insufficient. Understanding all of the previously listed parameters and their interrelationships is also critical for developing better solution strategies. Continuous research, ongoing monitoring, analysis, and documentation of lake pollution-related data, as well as the creation of databases to preserve it, are unavoidable requirements. Research projects about the referred parameters should be performed cooperatively with studies on agriculture, food, biodiversity, etc. (Thushari and Senevirathna 2020; Le Moal et al 2019).

The physical branch of lake pollution mostly focuses on analytical and numerical analyses of internal lake dynamics. This can include changing water levels, flow rates of currents, and temperature distributions throughout the lake, caused by the wind. These dynamics can be used to explain pollutant transport (Hutter et al 2010). Also, the retention time or the water residence time of a lake can be found by using the water volume of the lake and the flow rates of currents going into and out of the lake. The water residence time is useful to reach the residence time of a pollutant in a lake (Dodds and Whiles 2020). This way, physics, and biochemistry need to collaborate for the research topics such as the transport of nutrients in lakes, changing biomass concentration, the time required to wash out a microbial species, and movement of water in the lake receiving effects of atmospheric events such as wind through the surface of the lake (Hutter et al 2010; Dodds and Whiles 2020).

Because environmental and anthropogenic issues are inextricably linked, the development of multidisciplinary models that can encompass more than one parameter based on long-term observation data is a global priority. (Silva et al 2021; Sasakova et al 2018). Standardised and detailed models, considering different water sources (lakes, rivers, oceans, etc.), different pollution types (plastic, heavy metals, eutrophication, etc.), and different regional and natural conditions, remain deficient, and continuous research for sustainable strategies is needed. Solution options must be feasible in light of potential social, cultural, and economic constraints (Yang et al 2021; Kakade et al 2021). The resulting data of each precaution or treatment must be collected. This data can be used to analyze the effectiveness of each water pollution-related parameter, evaluate the current and future status of recovery, and introduce enhanced or new international solution strategies, for instance, Kentin ’s publication (2018) about microplastic restrictions in the European Union.

As preliminaries of complex solution strategies, numerical studies on dynamic systems, such as ecological and environmental systems, are widely examined by researchers in order to obtain an exhaustive understanding of the approximation of the unknowns in the system. Besides, the system is examined comprehensively including initial and boundary values and other parameters. As a result, multiple scientists have recently presented numerical methods to solve similar situations, i.e. Stanimirović et al (2022); Farsi (2022); Zhao and Hon (2022); Gürbüz et al (2022) and Ali et al (2022). In particular, numerical studies on lake pollution modelling and its numerical approach have been developed: Aguirre and Tully (1999); Varekamp (2003); Biazar et al (2006); Merdan (2009); Yüzbaşı et al (2012); Benhammouda et al (2014); Sokhanvar and Yousefi (2014); Hatipoğlu (2021), respectively. On the other hand, the complexity of nature can be understood by modeling and prediction of gene-expression patterns which acknowledging the role of the environment. The researchers also consider the Operational Research studies for the environmental protection: Weber et al (2009c); Kropat et al (2010); Özmen et al (2017); Weber et al (2009b, 2009a, 2008a) and Weber et al (2008b). Upon the mentioned studies, we developed our alternative approach for the investigation of the lake pollution model regarding a collocation-based algorithm.

In this study, we get a hold of lake pollution research ventures by introducing an alternative numerical approach to the dynamical pollution problem of three interconnected lakes. We consider the numerical approach based on Taylor approximation and investigate the convergence results regarding the numerical solution of the lake pollution model including the initial conditions. Besides, we investigate this ecological model and simulate the dynamics of lake pollution considering flow rates and volumes.

## Mathematical model

In this study, a dynamic system of lake pollution model proposed by Hatipoğlu (2021) is described as

1 $$\begin{aligned} \nonumber \frac{du_{1}(t)}{d\, t}= & {} \frac{F_{21}}{V_{2}}u_{2}(t)+\frac{F_{31}}{V_{3}}u_{3}(t)+f(t)-\frac{F_{12}}{V_{1}}u_{1}(t)-\frac{F_{13}}{V_{1}}u_{1}(t),\\ \frac{du_{2}(t)}{d\, t}= & {} \frac{F_{12}}{V_{1}}u_{1}(t)+\frac{F_{32}}{V_{3}}u_{3}(t)-\frac{F_{21}}{V_{2}}u_{2}(t)-\frac{F_{23}}{V_{2}}u_{2}(t),\\ \nonumber \frac{du_{3}(t)}{d\, t}= & {} \frac{F_{13}}{V_{1}}u_{1}(t)+\frac{F_{23}}{V_{2}}u_{2}(t)-\frac{F_{32}}{V_{3}}u_{3}(t)-\frac{F_{31}}{V_{3}}u_{3}(t), \end{aligned}$$

with the initial conditions

2 $$\begin{aligned} u_{1}(0)=\lambda _1 , \, u_{2}(0)=\lambda _2, \, u_{3}(0)=\lambda _3. \end{aligned}$$

In other words, the amount of pollutants in each lake is assumed to be constant at time zero. Following the ideas given by Hatipoğlu (2021), it is considered that initially, no pollutant exists in the lakes, namely $\lambda _i=0$ ($i=1,2,3$). Here $u_{1}$, $u_{2}$ and $u_{3}$ are the pollution amounts for each lake, respectively, at the time t, *f*(*t*) is the pollutant which comes in Lake 1, per unit time *t*, $V_i$ describes the volume of lake *i* and $F_{ij}$ is the flow rate from the lake *i* to *j* for $i,j=1,2,3$ and $a\le t\le b$. For example, $F_{21}$ represents the flow rate from lake 2 to lake 1. Here, the change in the amount of pollutant with respect to time in the first lake is governed by the inflow $F_{21}$ and $F_{31}$, and outflow $F_{12}$ and $F_{13}$. The pollution amounts for the lake 2 and lake 3 in system (1) can be similarly evaluated.

**Fig. 2:**
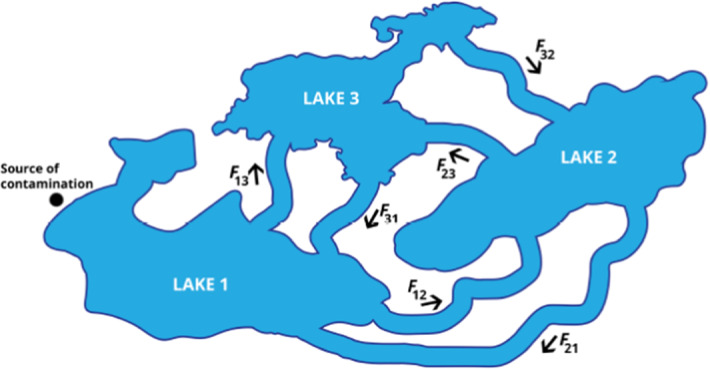
Illustration of the interconnected Lakes 1, 2, 3, and flow $F_{12}$, $F_{13}$, $F_{21}$, $F_{23}$, $F_{31}$, $F_{32}$ (Hatipoğlu 2021)

The origin of the system given in Eq. (1) is based on the mixture model presented by Aguirre and Tully (1999):

3 $$\begin{aligned} \frac{d M}{d t} = C_{in}(t) F_{in}(t) - C_{out}(t) F_{out}(t), \end{aligned}$$

where *M* represents the mass of the pollutant, F is the flow rate and *C* denotes the pollutant concentration (Aguirre and Tully 1999; Hatipoğlu 2021).

For the system given in (1), the constant volume of the lakes is introduced as

$$\begin{aligned} F_{12}= & {} F_{21}+F_{31}-F_{13}, \\ F_{23}= & {} F_{12}+F_{32}-F_{21}. \end{aligned}$$

Using the model given by (1) one obtains

4 $$\begin{aligned} \frac{d u_1}{d t}+\frac{d u_2}{d t}+\frac{d u_3}{d t}&= f(t),\nonumber \\ \frac{d}{d t}\left( u_1+u_2+u_3\right)&=f(t), \nonumber \\ u_1(t)+u_2(t)+u_3(t)&= \lambda _1+\lambda _2+\lambda _3+\int \limits _0^t f(t') d t'. \end{aligned}$$

Besides using the first equation of (1):

$$\begin{aligned} \left( \frac{F_{21}+F_{31}}{V_1}\right) u_1=\frac{F_{21}}{V_2} u_2+\frac{F_{31}}{V_3}u_3+f(t), \end{aligned}$$

leading to

$$\begin{aligned} u_1=\frac{V_1 \left[ V_3 F_{21}u_2+V_2 F_{31}u_3+V_2 V_3 f(t)\right] }{V_2 V_3 \left( F_{21}+F_{31} \right) }. \end{aligned}$$

Besides, from the second equation in model (1) and using the fact that $F_{21}+F_{23}=F_{12}+F_{32}$ the expression for $u_2$ is obtained as

$$\begin{aligned} u_2 = \frac{V_2 \left[ V_3 F_{12}u_1+V_1 F_{32} u_3\right] }{V_1 V_3 \left( F_{12}+F_{32}\right) }. \end{aligned}$$

Finally, the last equation in (1) leads to

$$\begin{aligned} u_3 = \frac{V_3 \left[ V_2 F_{13}u_1+V_1 F_{23}u_2\right] }{V_1 V_2 \left( F_{23}+F_{13}\right) }, \end{aligned}$$

where it is used that $F_{31}+F_{32} = F_{23}+F_{13}$. The steady states of the model (1) can be found using $d u_i/d t = 0$ for which each component can be obtained as

5 $$\begin{aligned} u_2= & {} \frac{-f(t) b_1}{a_1b_1+a_2 b_2-(a_3+a_4)b_3}, \end{aligned}$$

6 $$\begin{aligned} u_1= & {} \frac{b_3 }{b_1}u_2, \end{aligned}$$

7 $$\begin{aligned} u_3= & {} \frac{b_2 }{b_1}u_2, \end{aligned}$$

where $$b_1=a_3 (a_2+a_5)+a_4 a_5, b_2= a_4(a_1+a_6)+a_3 a_6, b_3=a_1(a_2+a_5)+a_2 a_6$$ with $$a_1=F_{21}/V_2, a_2=F_{31}/V_3, a_3=F_{12}/V_1, a_4=F_{13}/V_1, a_5=F_{32}/V_3, a_6=F_{23}/V_2$$.

## Numerical scheme

In this section, we introduce the numerical scheme for the solution of the problem (1)–(2). The notations for the method are given and a detailed explanation of the present method based on the Taylor series is presented. As a first step, we propose an approximation to the solution in the truncated Taylor series form

8 $$\begin{aligned} u_{i}(t)\cong & {} u_{i,N}(t) \nonumber \\= & {} \sum _{n=0}^{N}{u_{in}(t-c)^n}, \, \,\, i=1,2,3, \, n=0(1)N, \end{aligned}$$

where $t\in [a,b]$ and *N* is any positive integer. It is also defined by the degree of Taylor polynomial at $t=c$. Here we define the Taylor coefficients in the form

9 $$\begin{aligned} u_{in}=\frac{u_{i}^{(n)}(c)}{n!}, \, \, i=1,2,3. \end{aligned}$$

The approximation based on Taylor polynomials is constructed in detail (Gökmen and Sezer 2013). We consider the steps to obtain a numerical solution of the problem (1)–(2). This algorithmic approach is introduced at first by the matrix representation of the approximated solution which is suggested in (8).

10 $$\begin{aligned} u_{i}(t)= \textbf{T}(t)\textbf{A}_{i}, \, \, i=1,2,3, \end{aligned}$$

where

$$\begin{aligned} \textbf{T}(t)= & {} \left[ \begin{array}{ccccc} 1 &{} (t-c)&{} (t-c)^2 &{} \cdots &{} (t-c)^N \\ \end{array} \right] , \\ \textbf{A}_{i}= & {} \left[ \begin{array}{ccccc} a_{i0} &{} a_{i1}&{} a_{i2} &{} \cdots &{}a_{iN} \\ \end{array} \right] ^{T}, \, \, i=1,2,3. \end{aligned}$$

Thus we have the matrix relation to show the approximation. In our model, we also have derivatives of the unknown functions $u_1(t)$, $u_2(t)$, and $u_3(t)$, respectively (Gürbüz 2019). Therefore, we present the matrix form of the derivative of the unknowns as follows:

11 $$\begin{aligned}{}[u'_{i}(t)]= \textbf{T}(t)\textbf{B}\textbf{A}_{i}, \, \, i=1,2,3, \end{aligned}$$

where

$$\begin{aligned} \textbf{B}=\left[ \begin{array}{ccccc} 0 &{} 1 &{} 0 &{} \cdots &{} 0 \\ 0 &{} 0 &{} 2 &{} \cdots &{} 0 \\ \vdots &{} \vdots &{} \vdots &{} \ddots &{} \vdots \\ 0 &{} 0 &{} 0 &{} \cdots &{} N \\ 0 &{} 0 &{} 0 &{} \cdots &{} 0 \\ \end{array} \right] . \end{aligned}$$

Now we construct the matrix representation of the system (1). According to the previous matrix relations, we can build a matrix system for the dynamical system which is introduced in Sect. 3. Therefore, we consider the relations in (10) and (11) and we construct the following matrix relation for the $3 \times 3$ system as follows:

12 $$\begin{aligned} \textbf{u}'_{i}(t)- \textbf{F}_{ij}\textbf{V}_{i}\textbf{u}_{i}(t)=\textbf{f}(t), \, \, i,j=1,2,3. \end{aligned}$$

Here we define the following matrices for the unknown functions and their derivatives in (12):We also define the rest of the matrices in (12) for $i,j = 1,2,3.$

$$\begin{aligned} \textbf{f}(t) = \left[ \begin{array}{c} f(t) \\ 0 \\ 0 \\ \end{array} \right] , \, \textbf{F}_{ij} = \left[ \begin{array}{cccc} F_{21} &{} F_{31} &{} F_{12} &{} F_{13} \\ F_{12} &{} F_{32} &{} F_{21} &{} F_{23} \\ F_{13} &{} F_{23} &{} F_{32} &{} F_{31} \\ \end{array} \right] \, \text {and}\, \textbf{V}_{i} = \left[ \begin{array}{cccc} \frac{1}{V_{2}} &{} \frac{1}{V_{3}} &{} -\frac{1}{V_{1}} &{} -\frac{1}{V_{1}} \\ \frac{1}{V_{1}} &{} \frac{1}{V_{3}} &{} -\frac{1}{V_{2}} &{} -\frac{1}{V_{2}} \\ \frac{1}{V_{1}} &{} \frac{1}{V_{2}} &{} -\frac{1}{V_{3}} &{} -\frac{1}{V_{3}} \\ \end{array} \right] . \end{aligned}$$

Besides, we consider the matrix form of the initial conditions by using the procedure above. Thus we have13Now we define the augmented matrix by using the Eqs.  (10), (11), and (12) as follows:

14 $$\begin{aligned} \textbf{W}(t)\textbf{A}_{i}= \textbf{f}(t), \quad [\textbf{W}(t); \textbf{f}(t)]. \end{aligned}$$

Specifically, we consider $\det (\textbf{W})\ne 0$. Therefore we complete the matrix representations for the system in (1). Then we describe the collocation points as follows:

15 $$\begin{aligned} t_{l}=a+\frac{(b-a)}{N}l, \quad l=0,1,2,...,N. \end{aligned}$$

The collocation points (15) are now replaced into the Eq. (14). Accordingly, we define the fundamental matrix equation

16 $$\begin{aligned} \textbf{W}\textbf{A}_{i}=\textbf{f}. \end{aligned}$$

As a final step, we consider the new augmented matrix form by replacing the last three rows of the (16) with the matrix equations in (13).

17 $$\begin{aligned}{}[\tilde{\textbf{W}};\tilde{\textbf{f}}]. \end{aligned}$$

In this case, we have $\det (\tilde{\textbf{W}})\ne 0$. By solving the system in (17) with the help of the Gauss Elimination method, we obtain the unknown coefficients (Gürbüz and Sezer 2019). Consequently, we obtain the Taylor approximation for the numerical solution of the system (1) with the initial conditions in (2).

## Convergence

In this section, we consider the convergence of the present technique for the solution of the problem which is defined in (1)–(2). Namely, we describe the convergence of the numerical algorithm which we introduced in our study. The main idea is to describe the convergence rate of this technique; we measure it to obtain an efficient value between the solution point and the estimation that goes to zero. For the standard solution procedure of the problem, we apply the following statements:.

### Theorem 1

For any 3-dimensional dynamical system:

18 $$\begin{aligned} u_i(t)= & {} f_i(t,u_i(t)), \nonumber \\ u_i(0)= & {} \lambda _i, \, \, \, \, \forall \, \, i=1,2,3, \end{aligned}$$

we define an analytical function *g* which is of mapping $g^3: \mathbb {R}^3 \rightarrow \mathbb {R}^3$. It is also shown as

19 $$\begin{aligned} g^1(t)= & {} f_i(t,u_i(t)), \nonumber \\ g^2(t)= & {} D g^1(t) f_i(t,u_i(t)), \\ g^3(t)= & {} D g^2(t)f_i(t,u_i(t)), \, \, \, \, \forall \, \, i=1,2,3, \nonumber \end{aligned}$$

where *D* is the derivative of the functions $g^1(t)$ and $g^2(t)$, respectively. Therefore, we define the function by the series expansion as

20 $$\begin{aligned} u_i(t)=\lambda _i+\sum _{n=1}^{\infty } \frac{1}{n!}g^n(\lambda )(t-c) \, \, \, \, \forall \, \, i=1,2,3, \end{aligned}$$

which is of the unique solution to the problem (1)–(2). Thus we have any solution of the system

$$\begin{aligned} u_n(t)=g^n(t), \, \, \, \, \forall \, \, n\ge 1. \end{aligned}$$

### Proof

(Blanchard et al 2012).

### Theorem 2

Let us consider the system in (18) where $f_i(t,u_i(t))$ is continuously differentiable for all $i=1,2,3$. Thus there exists an $\varepsilon >0$ such that any solution of the system is unique in the interval $[-\varepsilon , \varepsilon ]$. Therefore we assured the analytical solution of any 3-dimensional system.

### Proof

(Zachmanoglou and Thoe 1986).

### Theorem 3

Let us consider the system in (18). Suppose that for any $d>0$, $f_i(t,u_i(t))$ is defined as analytic at [0, *d*]. Then there exists a unique solution of the system $u_i(t)$ for $i=1,2,3$ with the initial conditions (2). Therefore, $u_i(t)$ is analytic near 0 (Alsaker 2009; Driver 2003).

### Proof

The proof of theorem for the *n*-dimensional system is introduced in detail (Alsaker 2009; Driver 2003).

### Lemma 1

Let us consider the system in (18). Suppose $h_i$ is analytic near $0\in [0,d]$ for $i=1,2,3$. There exists

$$\begin{aligned} h_i \ll \frac{Cr}{r-z_1- z_2- z_3}, \end{aligned}$$

where *C* and *r* are constants and $z_1, z_2, z_3\in \mathbb {C}$.

### Proof

(Alsaker 2009; Driver 2003).

### Theorem 4

Let *f*(*x*) be a function defined on the interval [*a*, *b*]. Consider that $F_{i,j}$, $i, j=1,2,3$ are continuous and differentiable functions defined in [*a*, *b*], $u_{i}$, $i=1,2,3$ are the exact solutions of the problem (1)–(2), and $u_{i, N}$ are the approximate solutions obtained by the *N*-th order of Taylor series; then

$$\begin{aligned} \Vert u_{i}-u_{i,N}\Vert _{\infty }\le \frac{\max _{a\le t\le b} |(t-c)^{N+1}|}{(N+1)!}|u_{i}^{N+1}(\zeta )|+\kappa \max _{0\le n\le N}|e_{i,n}(c)|\end{aligned}$$

where $\kappa =\Vert \ell _{i}\Vert _{\infty }=\max _{a\le t\le b}\{|l_{i,0}(c)|, |l_{i,1}(c)|,...,|l_{i,n}(c)|\}$, and $e_{i,n}(c)=u_{i}^{(n)}(c)-u_{i,N}^{(n)}(c)$ for all $a \le \zeta \le b$.

### Proof

We can show the following inequality by using the approximation $u_{N}(t)$ in Eq. (8) and the Taylor series $T_{i,n}(t)$,

$$\begin{aligned} \Vert u_{i}(t)-u_{i,N}(t)\Vert _{\infty }\le \Vert u_{i}(t)-T_{i,n}(t)\Vert _{\infty } \le \Vert T_{i,n}(t)-u_{i, N}(t)\Vert _{\infty } \end{aligned}$$

where $T_{i,n}(t)=\sum _{n=0}^{N}\frac{u^{(n)}_{i}(c)(t-c)}{n!}$ for $c \in \mathbb {R}$ and $t\in [a,b]$. Now, we use the remainder estimation theorem for the Taylor series approach around $t=c$Wang and Wang (2014):

21 $$\begin{aligned} \left|R_{i,n}(t) \right|\le \frac{u_{i}^{(N+1)}(\zeta )}{(N+1)!} . \max _{a\le t\le b} \left|(t-c)^{N+1}\right|= \frac{\max _{a\le t\le b} \left|(t-c)^{N+1}\right|}{(N+1)!} u_{i}^{N+1}(\zeta ), \end{aligned}$$

where $R_{i,n}(t)=u_{i}(t)-T_{i,n}(t)=\frac{u_{i}^{(N+1)}(\zeta )}{(N+1)!}(t-c)^{N+1}$. Then we have

22 $$\begin{aligned} \left|T_{i,n}(t)-u_{i,N}(t) \right|&= \left|\sum _{n=0}^{N} \left( u_{i}^{(n)}(c)-u_{i,N}^{(n)}(c)\frac{(t-c)^{n}}{n!}\right) \right|\nonumber \\&=\left|\xi _{i,n} . \ell _{i} \right|\le \Vert \xi _{i,n} \Vert _{\infty } . \Vert \ell _{i} \Vert _{\infty } \le \kappa \Vert \xi _{i,n} \Vert _{\infty }. , \end{aligned}$$

where

$$\begin{aligned} \xi _{i,n}=(e_{i,0}(c), e_{i,1}(c),..., e_{i,n}(c), ..., e_{i,N}(c)),\\ \ell _{i}=(l_{i,0}(c),l_{i,1}(c),...,l_{i,n}(c),...,l_{i,N}(c))^T \, \text {for}\,\\ e_{i,n}(c)=u^{(n)}_{i}(c)-u^{(n)}_{i,N}(c), \, \text {and}\, \, l_{i,n}=\frac{(t-c)^{n}}{n!}. \end{aligned}$$

Now, we consider (21) and (22) and we obtain

$$\begin{aligned} \Vert u_{i}(t)-u_{i,N}(t)\Vert _{\infty }&\le \frac{u^{(N+1)(\zeta )}}{(N+1)!}. \max _{a\le t\le b} \left|(t-c)^{N+1}\right|+ \Vert \ell _{i}\Vert _{\infty }.\Vert \xi _{i,n}\Vert _{\infty }\\&=\frac{\max _{a\le t\le b} \left|(t-c)^{N+1}\right|}{(N+1)!} \left|u_{i}^{(N+1)}(\zeta )\right|+ \kappa \max _{0\le n\le N} \left|e_{i,n}(c) \right|. \end{aligned}$$

Therefore, we acquire that the numerical solution of the problem (1)–(2) has the *N*-th rate of convergence and we have guaranteed the adequacy of the present algorithm.

## Numerical simulations

**Fig. 3:**
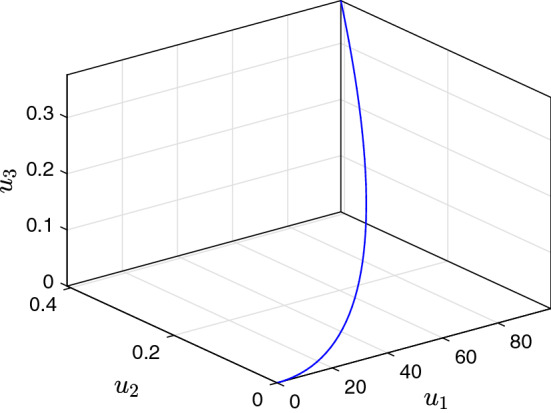
Phase plane diagram of the variables $u_1,u_2$ and $u_3$ for interconnected lakes. Parameters are given in Sect. 6

It is fundamental to test the method in terms of feasibility and efficiency. Therefore, we designed an exemplifying simulation. The parameters are selected as $F_{12}=24\, km^3/year$, $F_{13}=22\, km^3/year$, $F_{21}=14\, km^3/year$, $F_{23}=18\, km^3/year$, $F_{31}=32\, km^3/year$, $F_{32}=8\, km^3/year$, $V_{1}=2900\, km^3$, $V_{2}=850\, km^3$, and $V_{3}=1180\, km^3$ (Hatipoğlu 2021). The initial conditions are assumed as $u_{1}(0)=0$, $u_{2}(0)=0$, and $u_{3}(0)=0$. In Fig. 3, the phase plane analysis of the model (1) for three interconnected lakes is shown. Here, as demonstrated, the pollution amounts for all three lakes increase in time when the pollutant is imposed to the Lake 1 only once. This refers to a situation where the function for pollutant has a spike at time *t* (when the waste was dumped) and that is zero everywhere else. The simulation of the model in Fig. 3 is performed using MATLAB’s ode45 solver.

**Fig. 4:**
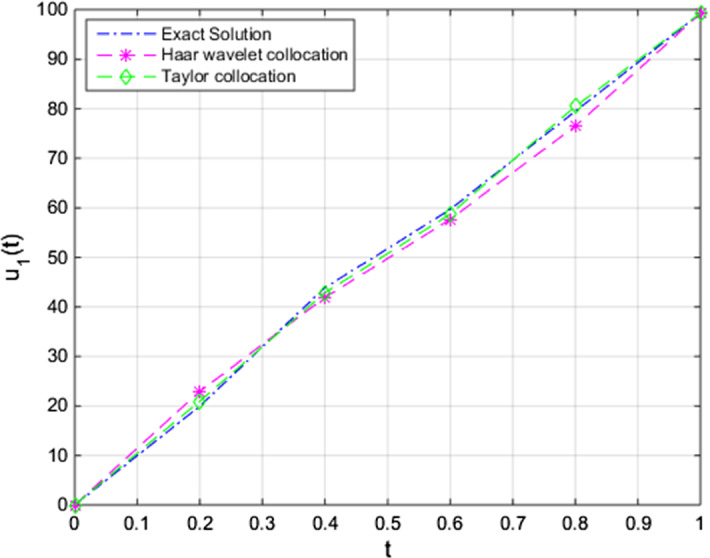
Comparison of the approximate Taylor and Haar wavelet collocations with the exact solution of $u_{1,N}(t)$ with $u_1(t)$ (pollution in Lake 1), for $N = 8$

**Fig. 5:**
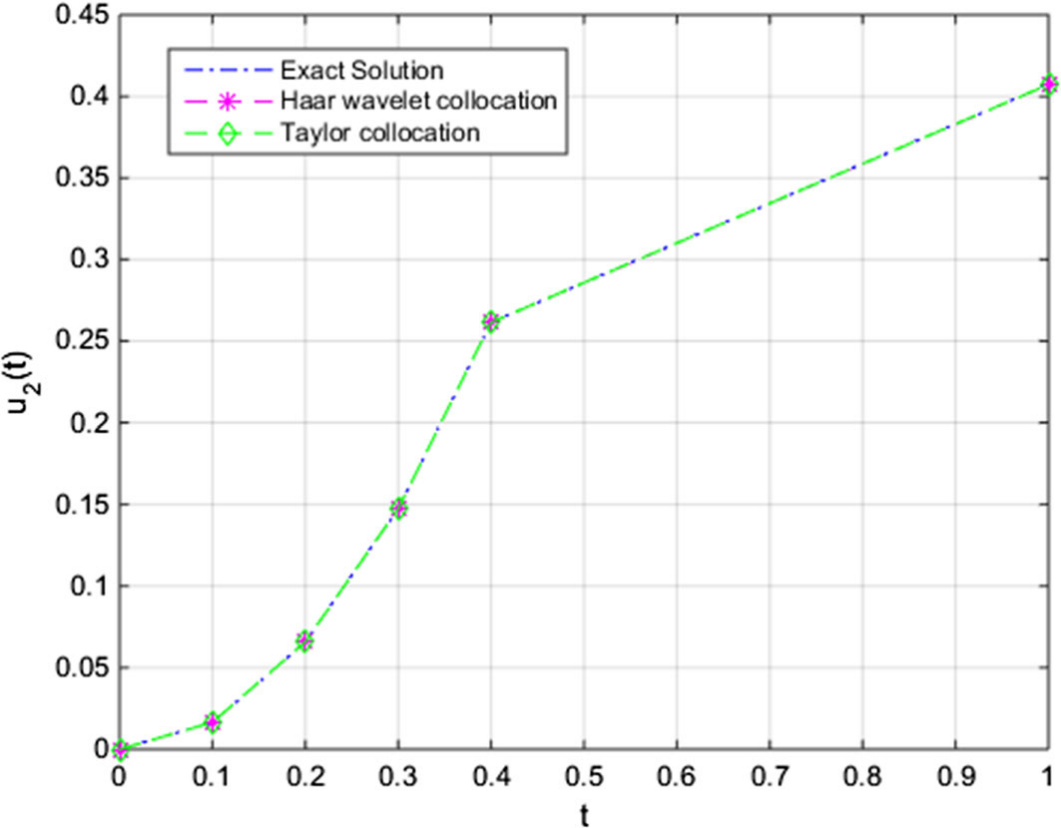
Comparison of the approximate Taylor and Haar wavelet collocations with the exact solution of $u_{2,N}(t)$ with $u_2(t)$ (pollution in Lake 2) for $N = 8$

**Fig. 6:**
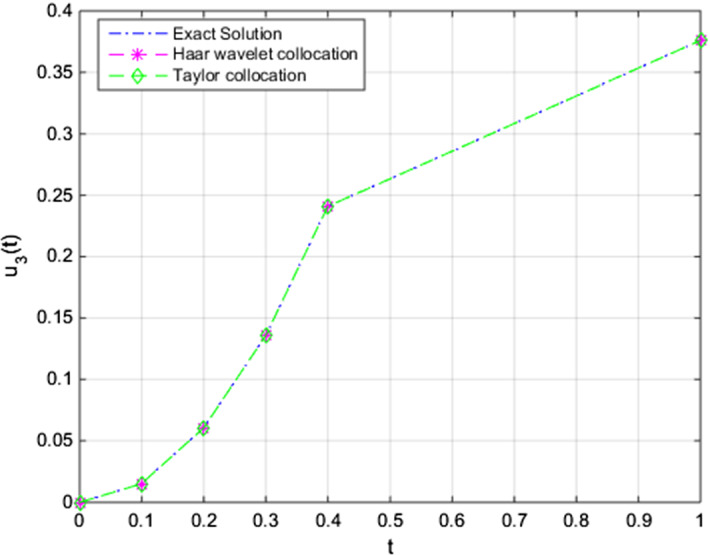
Comparison of the approximate Taylor and Haar wavelet collocations with the exact solution of $u_{3,N}(t)$ with $u_3(t)$ (pollution in Lake 3) for $N = 8$

Here, by taking *f*(*t*) as 100, approximate solutions are achieved. Finally, we compared solutions of our numerical method, exact solution, and solutions of the Haar wavelet collocation method. In Figs. (4), (5), and (6), the comparisons for different initial conditions ($u_{1}(0)$, $u_{2}(0)$, and $u_{3}(0)$, respectively), are given.

We obtain the outcomes with a suitable choice of parameters regarding our present algorithm. The findings of the application of the technique show that the numerical results have a better approximation than the previously studied numerical approaches for the unknown functions of time, $u_{1}(t), u_{2}(t)$, and $u_{3}(t)$, respectively, in the system. The efficiency and robustness of the technique are also supported by the findings in Sect.  5. In Fig. 4, we can obtain the pollutant amount of the first lake on time interval [0, 1]. Figures  5 and 6 have beneficial results concerning the numerical algorithm results and we have approximate solutions to the present problem under given initial conditions and approach the exact solutions. We have applied the numerical method for $N=8$ and for further iterations we acquire an effective outcome of the present method. Therefore, we can easily see that the method is of efficient testing results.

As we can understand from the figures of each numerical solution, we obtain approximations that have advantageous results close to the initial conditions. Then it is easily seen that the approaches move slightly and approximation grows stronger over time.

## Conclusion

Limited access to fresh water sources is becoming a global issue, and pollution, as humanity’s greatest burden, has received significant attention in the past decade. Observing and analysing the contamination in water sources are crucial steps for a safe environment. In this context, mathematical models provide a better understanding of the water environment to investigate pollution levels over time and may give hints to improve the water quality.

In this paper, we revisit an example three-components lake pollution model, where a constant ratio of pollutants is taken into account for the first lake, e.g. $f(t)=100$. The lakes are assumed to be well mixed with a single direction of flow. The relationship between all three lakes has been described using the preliminary mathematical analysis. Then, an alternative numerical approach, based on the Taylor series, is introduced for the solution of the problem. Besides, the numerical comparisons of the model (1) in terms of exact and Haar wavelet collocation methods have been demonstrated for a fixed contamination source. The results of this comparison show an excellent agreement between the proposed system and its numerical implementation, implying a successful application of the method to the differential equation system in model (1).

Environmental issues are critical in this day and age. There are many recent mathematical studies considering various prospects of environmental issues including the structure of the system, and the type of pollution using diverse numeric applications. For instance, Daily ’s thesis (2021) investigates various mathematical models of plastic pollution in lakes using two- and three-dimensional plastic transport mechanisms. Song and Pang (2021) studied a lake environment in narrow and generalised mathematical aspects and also connected their research with economical loss and benefit. Shiri and Baleanu (2021) modelled pollution amounts in one lake and multiple lakes connected to a river, using a system of fractional differential equations; they also applied explicit and implicit methods and compared local and global errors. Using experimental data about heavy metal pollution in small lakes, Geng et al (2022) proposed a numerical model based on the lattice Boltzmann method that can help to predict a theoretical basis for heavy metal treatment. These are just examples to show the variety, abundance, and up-to-dateness of studies related to lake pollution. Furthermore, Ghosh et al 2021 have investigated a system of three lakes with interconnecting channels (Biazar et al 2006), highly similar to the system (Hatipoğlu 2021) used in this study, applying the new iterative method (NIM, also known as DJM) (Daftardar-Gejji and Jafari 2006). Additional numerical methods, like the ones analyzed in mentioned research papers, can be applied to the same dynamical system and compared as a follow-up study.

The work presented in this paper can be extended in a couple of directions. First, the stability of the system (1) can be determined through linearisation, and the change in the dynamics of all three lakes can be determined with respect to specific system parameters to better understand the sensitivity of the model to various flow rates and volumes. Second, the contamination from one lake to another does not occur instantaneously and thus requires some time delay. Therefore, it is more plausible that constant delay terms are considered during the pollution of the lakes. Since the numerical method implemented in this paper is reliable, it provides wide applicability including real-world applications for multiple lakes with interconnected channels. Furthermore, prediction models are frequently used and improved day by day in weather sciences. For example, deep learning models are already used in short-term and accurate precipitation forecasting (Ravuri et al 2021). Efforts on pollution prediction have already started as shown by Dighe et al ’s software simulation (2022) of Aguirre and Tully ’s lake pollution model (1999). When considering the interconnectivity of life’s problems, applications of deep learning models to water pollution prediction may be immensely beneficial.

In summary, the world is struggling with the interlinked threats of climate change (Ford et al 2022), outbreaks (Yang et al 2021), natural and humanity-sourced disasters (Fang et al 2018), and various pollutions. Even though there is not yet a model to evaluate all these interrelated problems, there are good examples for evaluation of co-occurrences, such as Ford et al ’s study (2022) on climate change and coastal plastic pollution or Marazziti et al ’s study (2021) on climate change, air pollution, COVID-19 pandemic, and mental health. Even though it is overwhelming to model and solve all of Earth’s problems, the modelling of this vast and extremely complex system and future predictions would make life extraordinarily easy and safe. However, this might only be possible in science fiction, at least for the moment. Under these circumstances, each little contribution has an impact to create a better future. Here, we focus on lake pollution among all environmental issues due to the importance of lakes, comprising a big portion of water resources, for domestic use, health, economy, etc.

In conclusion, our alternative numerical approach, modelling the pollution problem in a dynamical system of three interconnected lakes, results in highly accurate information about the amount of pollution through time and constitutes an essential source of knowledge for scientists and researchers working in this field. The novelty of the study is about further numerical investigation by using the modified technique on a dynamical system problem. By this approach, we have a better understanding of the analysis of the present model which includes stability. The convergence results of the numerical approach explain the robustness of the technique. The alternative aspects of the work present how the numerical investigation gives a detailed answer for the model and opens future questions in the field. As a future outlook, the technique can be applied to stochastic models which may lead to open further discussions on biophysical problems Farah et al (2022). Also, the application of this approach to other pollution types, water source systems, or ecological issues with the collection and integration of related data would contribute to the understanding of the environmental matters for solving these issues or taking precautions.

## Data Availability

The datasets generated during and/or analysed during the current study are available from the corresponding author on reasonable request.
